# Association between elevated adiponectin level and adverse outcomes in patients with heart failure: a systematic review and meta-analysis

**DOI:** 10.1590/1414-431X20198416

**Published:** 2019-07-15

**Authors:** Wenwei Bai, Jingjing Huang, Min Zhu, Xiaoyong Liu, Jianping Tao

**Affiliations:** 1 Department of Cardiology, The Second Affiliated Hospital of Kunming Medical University, Kunming, Yunnan, China; 2 Department of Anesthesiology, The Second Affiliated Hospital of Kunming Medical University, Kunming, Yunnan, China

**Keywords:** Adiponectin, Heart failure, Mortality, Readmission, Meta-analysis

## Abstract

Studies regarding the prognostic value of circulating adiponectin level in patients with heart failure are conflicting. The aim of this meta-analysis was to evaluate the association between elevated circulating adiponectin level and adverse outcomes in patients with heart failure. We searched PubMed and Embase databases from their inception to June 2018. Original observational studies that investigated the prognostic value of adiponectin in heart failure patients and reported all-cause mortality or combined endpoints of death/readmission as outcome measure were included. Pooled risk ratio (RR) with 95% confidence intervals (CI) were estimated by higher versus lower circulating adiponectin level. A total of 7 studies involving 862 heart failure patients were identified. Meta-analysis showed that heart failure patients with higher adiponectin level had significantly increased risk of all-cause mortality (RR 2.05; 95%CI 1.22–3.43) after adjustment for potential confounders. In addition, higher adiponectin level was associated with an increased risk of the combined endpoints of death/readmission (RR 2.22; 95%CI 1.38–3.57). Elevated baseline circulating adiponectin level is possibly associated with an increased risk of all-cause mortality and the combined endpoints of death/readmission in patients with heart failure. Determination of circulating adiponectin level has potential to improve risk stratification in heart failure patients.

## Introduction

Despite substantial improvements in medical care, heart failure remains a worldwide public health concern with unacceptably high risk of morbidity and mortality (1). In developed countries, heart failure affects an estimated 1–2% of the adult population and 10–20% of people over 70 years of age (2). Heart failure is also a frequent cause of rehospitalization (3,4) and subsequently increases the health care costs. Therefore, early risk prediction of these patients is urgently needed for appropriate therapeutic decision making.

Biomarkers are frequently used for estimating prognosis in heart failure patients (5). Adiponectin, an adipocyte-specific cytokine, exhibits anti-inflammatory property and protective effects against obesity-related diseases (6). Circulating adiponectin level was increased in patients with heart failure irrespective of etiologies (7–9), implying substantial potential as a prognostic biomarker. However, adiponectin appears to lose its cardio-protective and anti-inflammatory properties in heart failure patients (10). Accordingly, several but not all studies (11,12) have reported that elevated adiponectin level emerged as an independent risk factor of adverse prognosis. Nevertheless, the magnitude of the prognostic value varied among these studies.

To our knowledge, no prior meta-analysis has examined the prognostic role of adiponectin level in patients with heart failure. Hence, we undertook a meta-analysis to evaluate the association between elevated circulating adiponectin level and adverse outcomes in heart failure patients.

## Material and Methods

### Data sources and literature searches

We made a comprehensive literature search of PubMed and Embase databases from their inception to June 2018 using the following search items: “adiponectin” AND “heart failure” AND “rehospitalization” OR “readmission” OR “mortality” OR “death” OR “prognosis” OR “major adverse cardiac events”. In order to identify any additional studies, we manually searched the reference lists of relevant reviews and included studies. There was no language restriction in the literature searches.

### Study selection

Original articles satisfying the following criteria were included: 1) observational studies enrolling heart failure patients; 2) baseline circulating adiponectin level as exposure; 3) all-cause mortality or combined endpoints of death/readmission as one of the endpoints; and 4) at least reported age-adjusted risk estimate of the prognostic value for the higher versus the lower adiponectin level. Studies were excluded if: 1) review articles, editorial, letter or conference abstract; 2) unadjusted risk estimate; and 3) reported risk estimate by continuous adiponectin level.

### Data extraction and quality assessment

All data were extracted by two independent authors using a standardized extraction form. Discrepancy was resolved by consensus. Data extracted included the first author's surname, year of publication, country of study, study design, sample sizes, percentage of men, age range or mean age, proportion of men, type of heart failure, adiponectin cutoff value, event number, fully adjusted hazard ratio (HR) or risk ratio (RR) with 95% confidence intervals (CI), follow-up duration, and adjusted confounders. The methodological quality of the selected studies was examined using the Newcastle-Ottawa Scale (NOS) (13). Studies with a rating of >7 points were deemed as having good quality.

### Data synthesis and analysis

Multivariable-adjusted risk estimates reported in each study were used in the meta-analysis. We pooled the RR with 95%CI for higher versus lower adiponectin level. Statistically significant heterogeneity across studies was quantified using I^2^ statistic >50% and Cochrane Q test P<0.10. A random effect model was selected when a statistically significant heterogeneity was observed; otherwise, we applied a fixed effect model. Sensitivity analysis was conducted by sequentially removing any single study at each turn to explore the reliability of the pooled risk estimate. We evaluated publication bias by the Egger linear regression test (14) and Begg's rank correlation (15). All data were analyzed using STATA version 12.0 (StataCorp, USA).

## Results

### Search results and study characteristics

**Figure 1.**
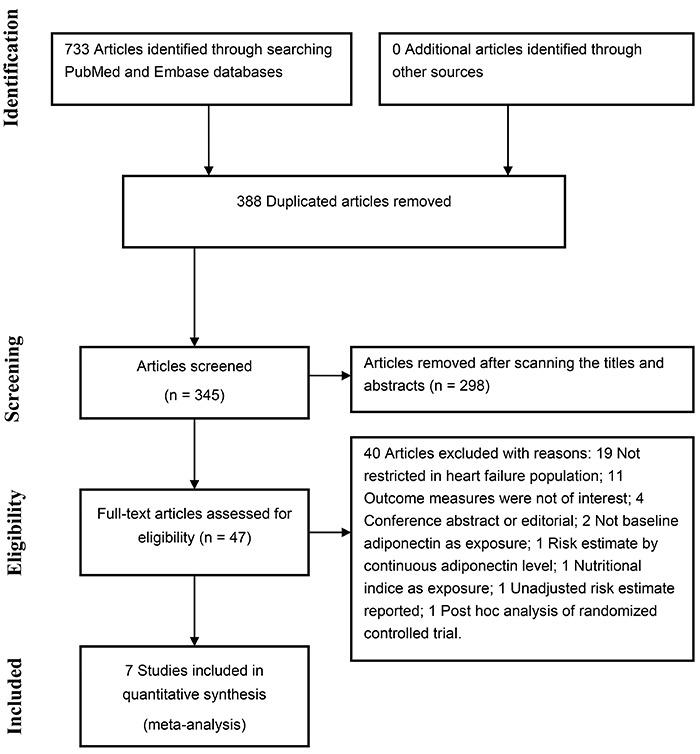
Flow chart of the study selection process.

Briefly, 345 potentially relevant articles were identified after duplicated records were removed. Of those, 298 articles were removed after scanning the titles and abstracts. The remaining 47 full-text articles were retrieved for detailed evaluation. Forty articles were further excluded for various reasons (Figure 1). Thus, 7 studies (11,12,16–20) were ultimately included in the meta-analysis.

The main characteristics of the included studies are summarized in Supplementary Table S1. Sample sizes ranged between 54 and 195 with a total of 862 heart failure patients. These studies were published between 2005 and 2014. Five studies (11,12,16,17,19) had a cohort design and two studies (18,20) had case-control designs. Follow-up duration ranged from 288 days to 7 years. For the assessment of methodological quality, the NOS scores of these studies ranged from 6 to 8 points (Supplementary Table S2).

### All-cause mortality

**Figure 2.**
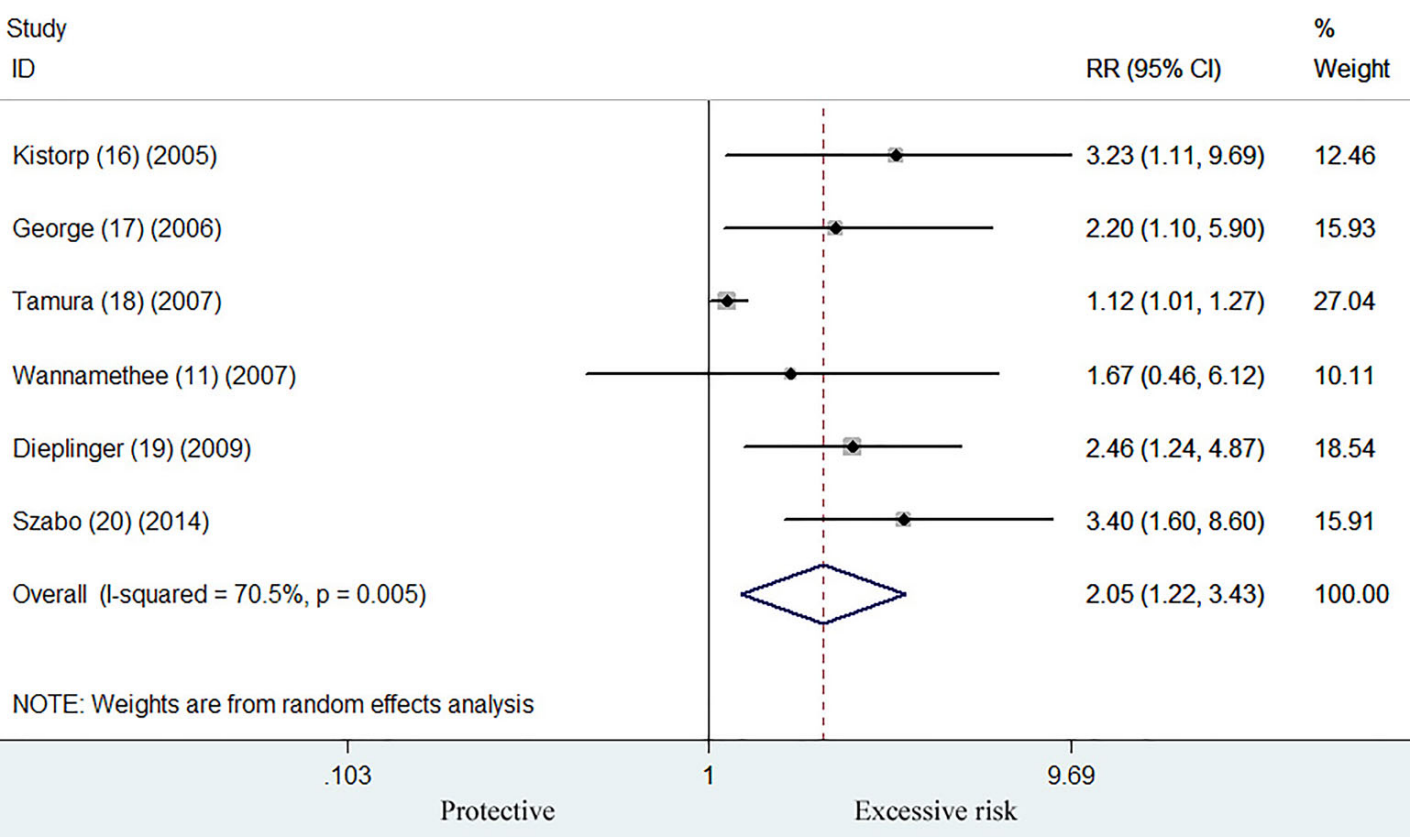
Forest plot showing risk ratio with 95%CI of all-cause mortality for the higher versus lower category of adiponectin level in a random effect model.

Six studies (11,16–20) were used to estimate the prognostic value of elevated adiponectin level on all-cause mortality. As shown in Figure 2, the pooled RR was 2.05 (95%CI 1.22–3.43) for the higher versus lower adiponectin level in a random effect model. Significant heterogeneity was observed across these studies (I^2^=70.5%, P=0.005). Publication bias was not detected by the Begg's (P=0.707) and Egger's tests (P=0.742). Sensitivity analyses showed that no influential changes were found with the omitting of any single study at a time (data not shown).

### Combined endpoints of death/readmission

**Figure 3.**
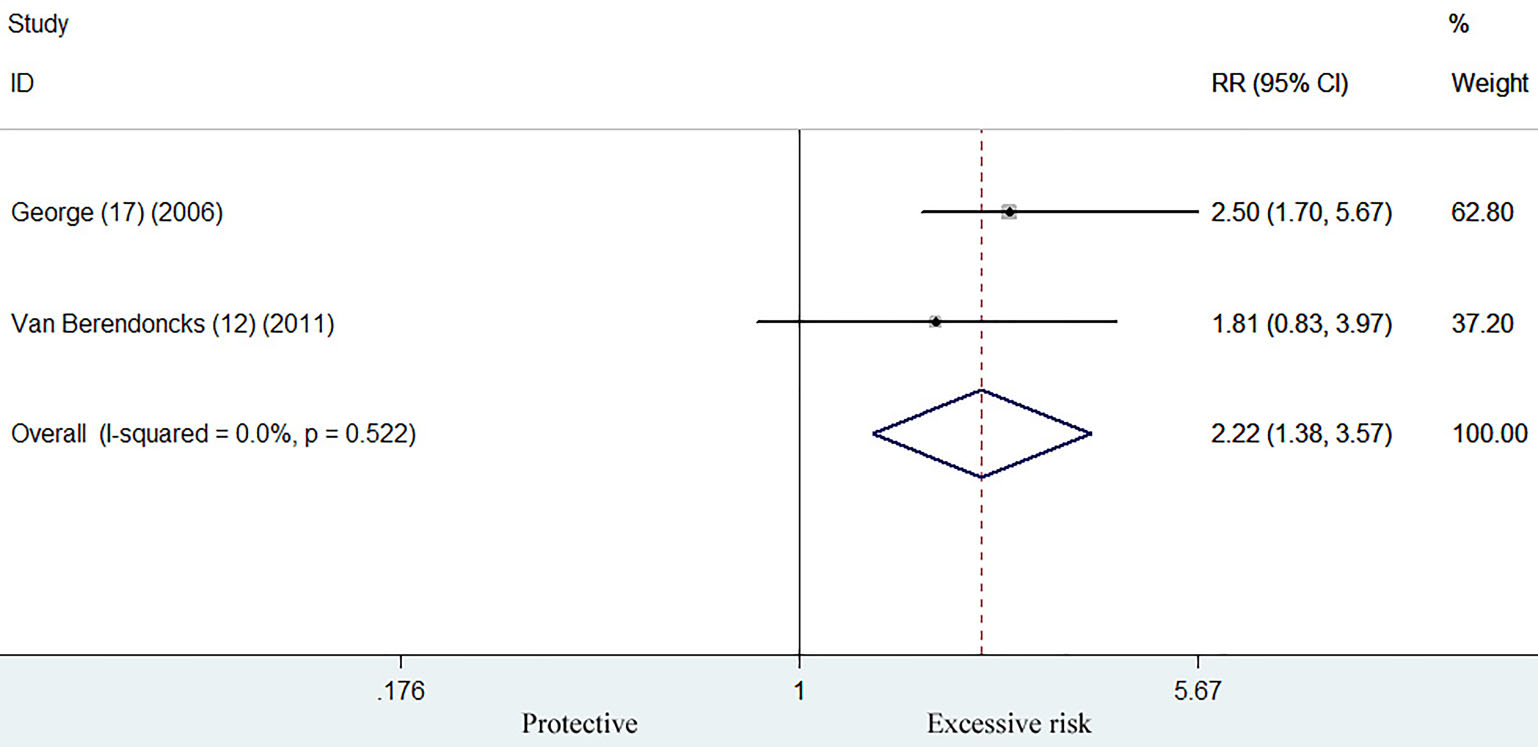
Forest plot showing risk ratio with 95%CI of combined end points of death/readmission for the higher versus lower category of adiponectin level in a fixed-effect model.

Two studies (12,17) reported combined endpoints as an outcome measure. As shown in Figure 3, heart failure patients with higher adiponectin level had significantly increased combined endpoints of death/readmission (RR 2.22; 95%CI 1.38–3.57) in a fixed-effect model. There was no significant heterogeneity across studies (I^2^=0.0%; P=0.522).

## Discussion

This meta-analysis demonstrated that elevated circulating adiponectin level was possibly associated with an increased risk of all-cause mortality and the combined endpoints of death/readmission in acute or chronic heart failure patients. Heart failure patients with the higher circulating adiponectin level had approximately 2.05-fold and 2.22-fold greater risk of all-cause mortality and the combined endpoints of death/readmission, respectively. Determination of adiponectin level may improve the risk stratification of heart failure patients.

The prognostic value of baseline adiponectin in heart failure patients was also supported by the continuous variable analysis. A *post hoc* analysis of GISSI-HF trial (21) indicated that per 1 SD increase in baseline plasma adiponectin level was independently associated with increased mortality (hazard ratio 1.24; 95%CI 1.12–1.37). Collectively, these results highlight that circulating adiponectin level plays an important role in risk stratification of heart failure patients. However, conflicting results exist. The prospective study of Berezin et al. (22) showed that per 3.5 μg/mL increase in adiponectin level was not significantly associated with composite endpoint of death or chronic heart failure hospitalization.

A previous meta-analysis concluded that higher adiponectin was associated with increased mortality in patients with already established cardiovascular disease (23). However, that meta-analysis was based on heterogeneous patients. By contrast, our meta-analysis focused on heart failure patients. Findings from our meta-analysis supported the baseline adiponectin level as a prognostic biomarker in patients with heart failure. Moreover, chronic heart failure patients with a high level of adiponectin after a 3-month treatment had a 3.8-fold increased risk of major adverse cardiac events (24). Accordingly, decrease in serum adiponectin level in response to treatment could predict a better prognosis in acute decompensated heart failure (25)

Cachexia or obesity could affect circulating adiponectin level. Adiponectin level was negatively correlated with obesity, but was also affected by age and gender (26). Increased adiponectin level could be caused by cardiac cachexia irrespective of body mass index in heart failure patients (27). Elevated adiponectin level may be a marker for wasting. Heart failure patients with cachexia have shown a poor prognosis (28). Obesity is a predictor of improved prognosis in patients with heart failure and adiponectin may explain why the prognosis of heart failure is better in the obese (29). Nevertheless, increased adiponectin level may simply reflect the severity of disease. Therefore, the prognostic value of adiponectin could be confounded by cardiac cachexia.

Several potential limitations of the current meta-analysis should be acknowledged. First, adiponectin level was determined only at baseline but follow-up changes were not monitored. More serial determinations are warranted to avoid possible misclassification because of fluctuations of adiponectin level. Second, the included studies used different cutoff values of adiponectin level and the optimal threshold of adiponectin level was not defined. Third, prognostic strength of the adiponectin level could be verified by a continuous variable analysis. However, we did not analyze the association between elevated adiponectin level and adverse outcomes by continuous data due to insufficient studies. Fourth, the impact of drugs on the circulating adiponectin level should be considered. Use of β-blockers and anti-heart failure agents could affect circulating adiponectin level (12,30). Lack of adjustment for these agents may have underestimated or overestimated the prognostic value. Finally, there was significant heterogeneity in the pooling of all-cause mortality outcomes. Different study designs, follow-up duration, cutoff values of adiponectin level, types of heart failure, and patients' characteristics, such as age and ejection fraction, may have partly contributed to the significant heterogeneity.

In conclusion, elevated baseline circulating adiponectin level might be independently associated with higher risk of all-cause mortality and combined endpoints of death/readmission in heart failure patients. Future well-designed studies are required to confirm the findings of this meta-analysis. Measurement of adiponectin level in heart failure patients has potential to improve risk stratification.

## Supplementary Material

Click here to view [pdf].
